# Restoring Mouth Injured by Air-Chambers

**Published:** 1904-11

**Authors:** 


Restoring Mouth Injured by Air-Chambers.—When the roof of the mouth had been ruined by having the tissues drawn down, Dr. Atkinson made a rubber plate to accurately fit the parts. The hypertrophied parts were painted with a saturated solution of salicylic acid in alcohol. That was done once daily for three or four days; then some plaster was put in the plate, and the plate put in the mouth. That was continued from time to time, the plaster cores being changed at each setting, increasing in thickness until the roof of the mouth was gradually reduced to its proper condition. I remember one case he showed me where he had reduced nearly half an inch of hypertrophied tissue by that means in the course of a few weeks.—*Items of Interest.*

